# Increased Vulnerability to Atrial Fibrillation Is Associated With Increased Susceptibility to Alternans in Old Sheep

**DOI:** 10.1161/JAHA.118.002331

**Published:** 2019-12-04

In the article by Pearman et al, “Increased Vulnerability to Atrial Fibrillation Is Associated With Increased Susceptibility to Alternans in Old Sheep,” which published online November 21, 2018, and appeared in the December 4, 2018 issue of the journal (*J Am Heart Assoc*. 2018;7:e009972. DOI: 10.1161/JAHA.118.009972), a correction was needed.

The article license was listed as, “© 2019 The Authors. Published on behalf of the American Heart Association, Inc., by Wiley. This is an open access article under the terms of the Creative Commons Attribution‐NonCommercial License, which permits use, distribution and reproduction in any medium, provided the original work is properly cited and is not used for commercial purposes.” To comply with a funding mandate, the license has now been corrected to “© 2019 The Authors. Published on behalf of the American Heart Association, Inc., by Wiley. This is an open access article under the terms of the Creative Commons Attribution License, which permits use, distribution and reproduction in any medium, provided the original work is properly cited.”

The authors regret the error.

The online version of the article has been updated and is available at https://www.ahajournals.org/doi/10.1161/JAHA.118.009972

